# Supplementary material for: Atomic-level structure determination of amorphous molecular solids by NMR
Source: Nat Commun. 2023 Aug 23;14:5138. doi: 10.1038/s41467-023-40853-2 (PMC10447443; doi:10.1038/s41467-023-40853-2)
Supplement: Supplementary file 1 — Supplementary Information [file 41467_2023_40853_MOESM1_ESM.pdf]

Supplementary Information

Atomic-Level Structure Determination of Amorphous Molecular Solids by NMR

Cordova *et al.*

Supplementary Notes

Raw data statement. The NMR raw data are available from https://doi.org/10.24435/materialscloud:gk-51 in JCAMP-DX version 6.0 standard format and original TopSpin format, as well as the input files for the MD simulations, the MD snapshots extracted, formation energies of intermolecular complexes, and all scripts used to perform the data analysis. All data and scripts are available under the license CC-BY-4.0 (Creative Commons Attribution-ShareAlike 4.0 International).

Supplementary Methods

1D ^{1}H MAS experiment (Fig. 1f): A one rotor period rotor-synchronized spin echo sequence, for background suppression, was used for acquisition. Pre-saturation was applied prior to excitation. No weighting function was applied upon processing.

Supplementary Table 1: Experimental details of the 1D ^{1}H MAS experiment.

MAS rate (kHz)	VT (K)	^{1}H 90° RF (kHz)	d1(s)	TD	SW (kHz)	SI
100	278	312.5	5	4096	227.3	8192

1D DNP enhanced ^{13}C CPMAS experiments (Fig. 1b-1d): A conventional cross-polarization (CP)[1] sequence was used for the acquisition of the spectra presented in Fig. 1b and 1c with a contact time of 2.5 and 0.1 ms respectively. The short contact time promotes the detections of protonated carbon atoms. Pre-saturation was applied prior to excitation.

For Fig. 1d an editing experiment (CPPI)[2] that relies on phase inversion was used for acquisition. In this sequence a second short (40 μs) cross-polarization block right after the initial one inverts -CH_2 groups whereas nulls -CH groups and retains -C and -CH3 groups with a positive intensity.

For all spectra spinal-64 ^{1}H decoupling[3] with an rf of 71.4 kHz was applied during acquisition.

A Lorentzian line broadening of 150 Hz was applied upon processing.

Supplementary Table 2: Experimental details of the 1D ^{13}C CPMAS experiments.

Exp.	MAS rate (kHz)	VT (K)	^{1}H 90° RF (kHz):	d1(s)	TD	SW (kHz)	SI	Contact Power (kHz): $^{1}H/^{13}C$	Contact time (ms)
Fig. 1b	10	100	71.4	2	2048	100	81 92	54 / 70	2.5
Fig. 1c	10	100	71.4	2	2048	100	81 92	54/70	0.1
Fig. 1d	10	100	71.4	3	988	100	81 92	54/83.3	2.5

2D DNP enhanced ^{13}C-^{13}C INADEQUATE experiment (Fig. 1h): A rotor-synchronized *J*-based refocused INADEQUATE[4,5] sequence was used for acquisition.

A States-TPPI acquisition scheme[6] was used to obtain phase-sensitive two-dimensional spectra.

Spinal-64 ^1H decoupling[3] with an rf of 83.3 kHz was applied only during acquisition.

A Lorentzian line broadening of 300 Hz was applied upon processing to both dimensions.

Supplementary Table 3: Experimental details of the 2D ^{13}C-^{13}C INADEQUATE experiment.

MAS rate (kHz)	VT (K)	90° RF amplitude (kHz)	d1(s)	Number of FID points: F2/F1	SW (kHz): F2/F1	Size of real spectrum: F2/F1	J evolution time (μs)	Contact Power (kHz): $^1H/^{13}C$	Contact time (ms)
10	100	83.3	3	806/40	81.5/25	2048/256	8	71.5 / 59.8	4

2D ^1H-^1H DQ/SQ experiment (Fig. 1e): An eight-rotor period rotor-synchronized BABAxy16[7] sequence was used for acquisition. Pre-saturation was also applied prior to excitation.

A States-TPPI acquisition scheme[6] was used to obtain phase-sensitive two-dimensional spectra.

A Lorentzian line broadening of 100 Hz was applied upon processing to both dimensions.

Supplementary Table 4: Experimental details of the 2D ^1H-^1H DQ/SQ experiment.

MAS rate (kHz)	VT (K)	90° RF amplitude (kHz)	d1(s)	Number of FID points: F2/F1	SW (kHz): F2/F1	Size of real spectrum: F2/F1	DQ recoupling time (μs)
100	278	312.5	5	9090/200	227.2/33.3	16384/256	80

2D DNP enhanced ^{1}H-^{13}C HETCOR experiment (Fig. 1h and 1i): A DUMBO-HETCOR[8] sequence was used for the acquisition. An eDUMBO-1_{22}[8] element (32 µs at 71.5 kHz), applied during t_1, increases the resolution of the indirect dimension by averaging and therefore decoupling ^{1}H-^{1}H homonuclear dipolar couplings.

The rescaling of the indirect dimension, caused by the application of the DUMBO element, was done with the aid of the 1D 100 kHz ^{1}H MAS spectrum.

A States-TPPI acquisition scheme[6] was used to obtain phase-sensitive two-dimensional spectra.

Spinal-64 ^{1}H decoupling[3] with an rf of 83.3 kHz was applied during acquisition.

A Lorentzian line broadening of 200 Hz was applied upon processing to both dimensions.

Supplementary Table 5: Experimental details of the 2D ^{1}H-^{13}C HETCOR experiment.

Exp.	MAS rate (kHz)	VT (K)	90° RF amplitude (kHz)	d1(s)	Number of FID points: F2/F1	SW (kHz): F2/F1	Size of real spectrum: F2/F1	Contact Power (kHz): ^{1}H/^{13}C	Contact time (ms)
Fig. 1h	10	100	83.3	2	1024/128	100/52	8192/1024	83.3/59.8	0.1
Fig. 1i	10	100	83.3	2	1024/28	100/47	8192/1024	83.3/59.8	0.5

Supplementary Table 6: ^{1}H and ^{13}C chemical shifts and widths (Gaussian σ) of amorphous AZD4625. [a] Indicates widths that represent several overlapping resonances, thus should be considered as upper bounds to the linewidths. The ^{1}H and ^{13}C resonances were assigned using the experimental spectra of **Fig. 1** of the main text. Due to the amorphous character of AZD4625, the acquired spectra have broad lineshapes which reduce spectral resolution and often obscure the identification of peak maxima. However, we believe that the assignment presented here is accurate enough to be used for our further analysis. The assignment of C1 is uncertain due to the low signal-to-noise ratio of the INADEQUATE spectrum of the amorphous AZD4625. The assignment of the aliphatic carbon atoms was performed using the 1D CPMAS spectra and the INADEQUATE spectrum of a crystalline form (shown in **Supplementary Fig. 1**). The carbon chemical shifts were referenced using glycerol and the proton chemical shifts using L-histidine hydrochloride monohydrate.

Label	^{1}H Chemical Shift / Width (ppm)	13C Chemical Shift / Width (ppm)
1	7.6/-	114/-
2	6.7/1.0[a]	129.7/5.5[a]
3	-	166.6/5.9[a]
4	4.3/1.0[a]	50.8/3.1
5	5.1/1.0[a]	46.9/2.3
6	3.3/0.8	45.8/2.6
7	3.7/1.0[a]	54/2.7
8	1.3/1.0[a]	30.3/2.0
9	3.4/0.7	71.3/3.4
10	-	149.3/2.0
11	-	103.3/5.0[a]
12	-	161/3.9[a]
13	8.5/1.0[a]	154/3.7[a]
14	-	142.2/2.0
15	-	149/2.0
16	-	125.7/2.5
17	-	124.7/3.4[a]
18	-	108.4/3.6[a]
19	-	162.7/2.6
20	7.6/0.6	114.7/6.0[a]
21	7.6/0.6	132.4/5.4[a]
22	7.8/0.6	108.4/3.0
23	-	159.4/3.4[a]
24	0.8/0.6	17.7/2.3
OH	11.3/1.8	-

Computational details for the MD simulations. To model the amorphous structure of AZD4625, we carried out MD simulations on periodic amorphous cells. The atomic positions of a single molecule were first optimized at the B3LYP-D3/6-31G(d,p) level of theory[9-13] in gas phase using the Gaussian 16 revision C.01 program.[14] Optimized coordinates and CHELPG charges[15] were extracted from the optimization and used as input to generate amorphous cells. Materials Studio[16] together with the COMPASS-III force field[17] were used to create cubic amorphous cells (43*43*43 Å) of 128 molecules placed randomly and with identical conformations in eight replicates. These multiple replicas allow the generation of a diverse set of structures. PDB files of the amorphous cells were saved as input for the MD-step. The Desmond program (Schrödinger 2021-4)[18] was used for all MD simulations throughout the study employing the OPLS4 force field.[19] The systems were initially equilibrated for 1 ns using the canonical (NVT) ensemble first at 100 K and then at 298 K. The temperature was held constant using a Nosé-Hoover chain thermostat[20,21] with a relaxation time of 1.0 ps. A second equilibration was carried out for 22 ns using the isothermal-isobaric ensemble (NPT) at 298 K and 1 bar where the temperature and pressure were held constant using the coupled Martyna-Tobias-Klein method[22] with a relaxation time of 1.0 ps. Production simulations were carried out for 500 ns using the NPT ensemble at 298 K and 1 bar with the same settings as in the second equilibration. Electrostatic interactions were included with a 9 Å cutoff. Trajectories were collected every 100 ps. Models of the amorphous structure were obtained by extracting evenly spaced snapshots from the last 100 ns of each MD simulation. Since no inversion of the aromatic ring containing the OH group was observed during the MD simulations, five simulations were carried out with a starting angle between the aromatic planes around -90° and three were run with a starting angle around 90°. This explains the 5:3 ratio of negative and positive angles in **Fig. 3d**. The eight simulations (corresponding to 1,025,280 molecular environments) were assumed to fully sample the conformational and noncovalent interaction space of the molecule in the amorphous phase.

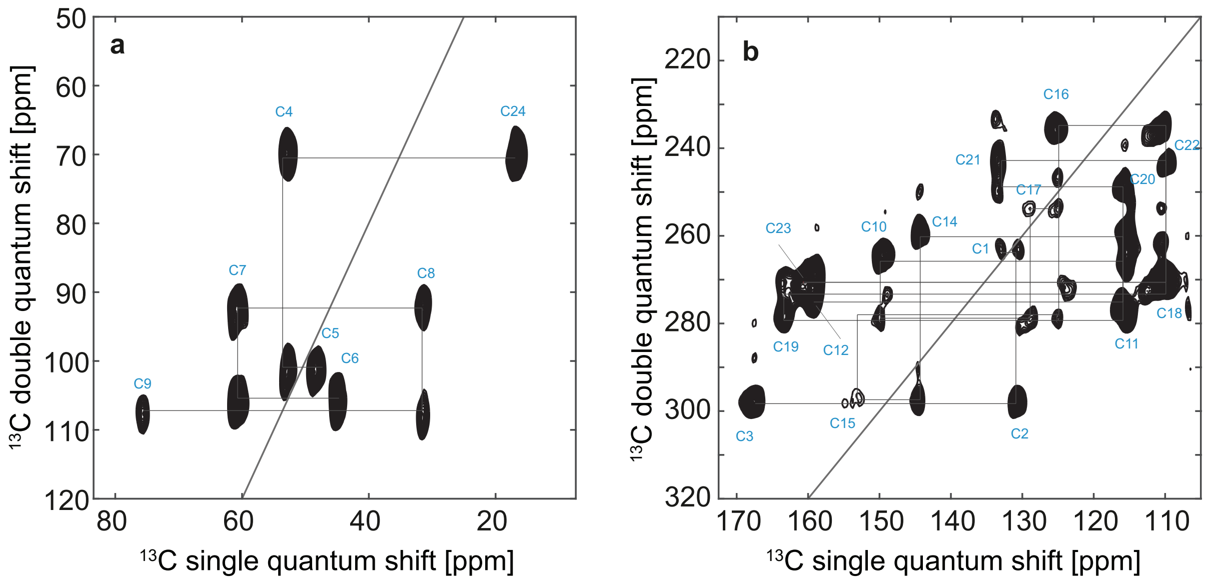

Supplementary Figure 1. ^{13}C-^{13}C INADEQUATE spectra of a crystalline form of AZD4625. In (**a**) the aliphatic and in (**b**) the aromatic regions are plotted.

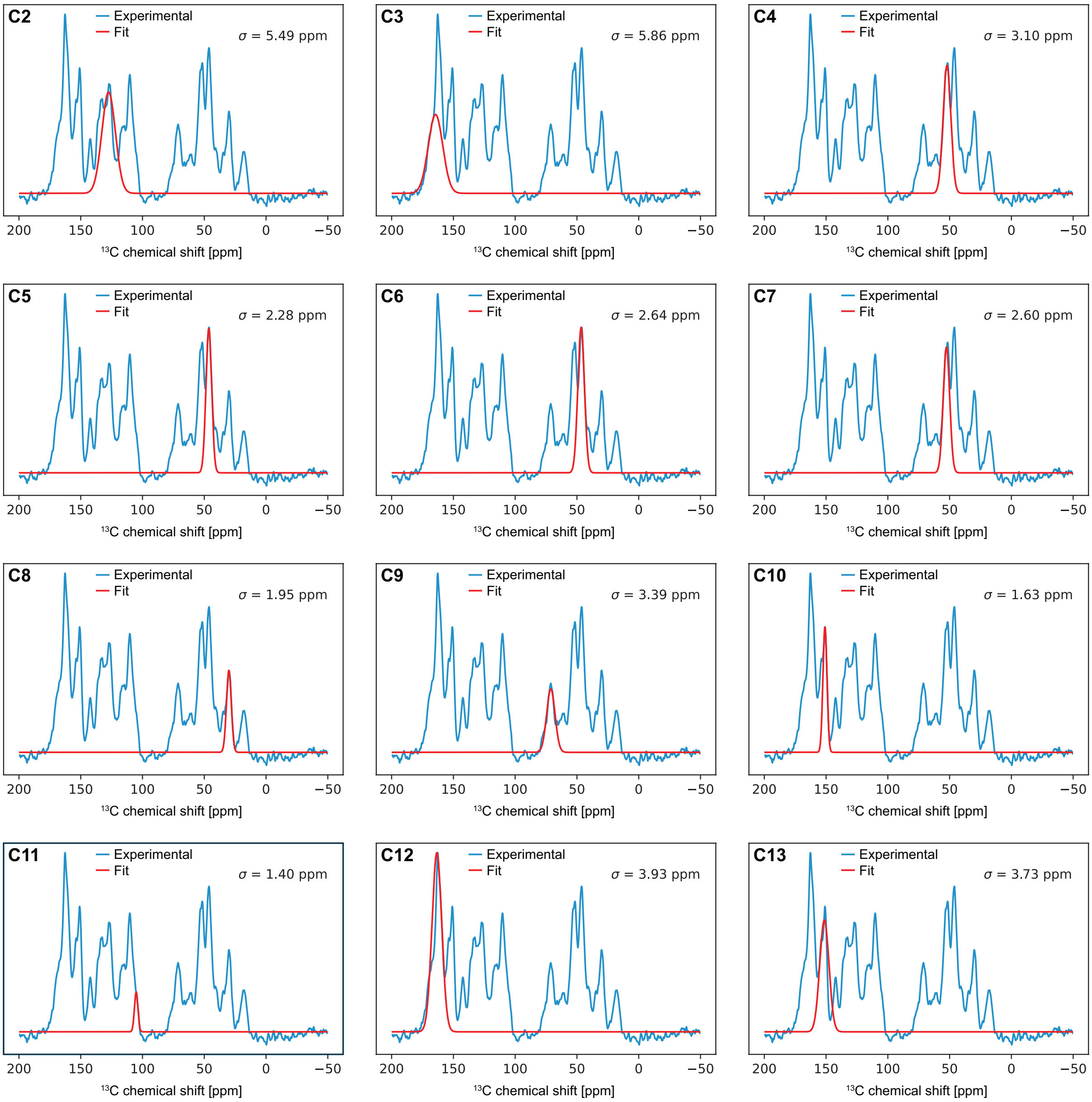

Supplementary Figure 2. Fits of individual resonances of C2-C13 from the 1D ^{13}C CPMAS NMR spectrum of amorphous AZD4625.

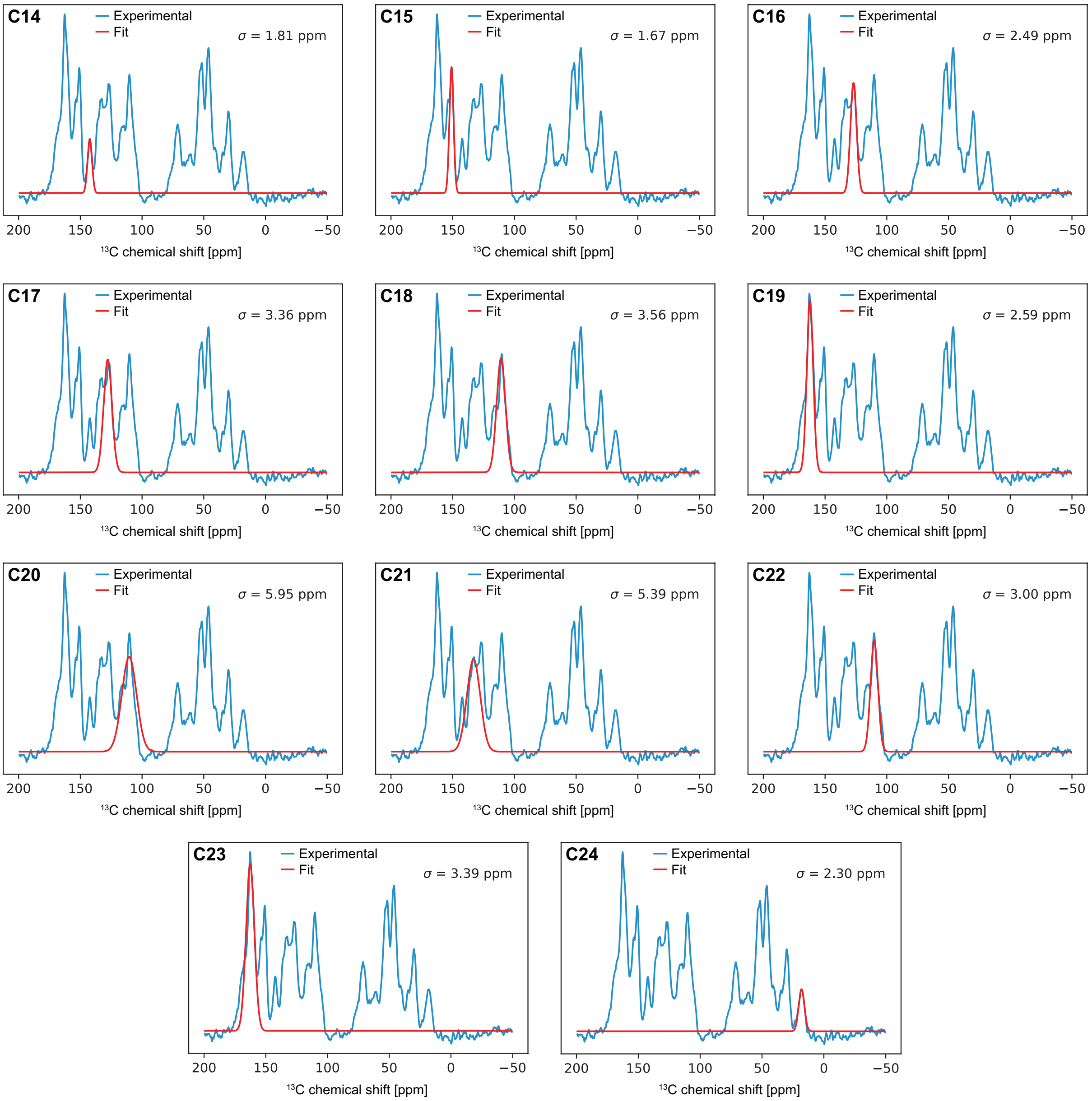

Supplementary Figure 3. Fits of individual resonances of C14-C24 from the 1D ^{13}C CPMAS NMR spectrum of amorphous AZD4625.

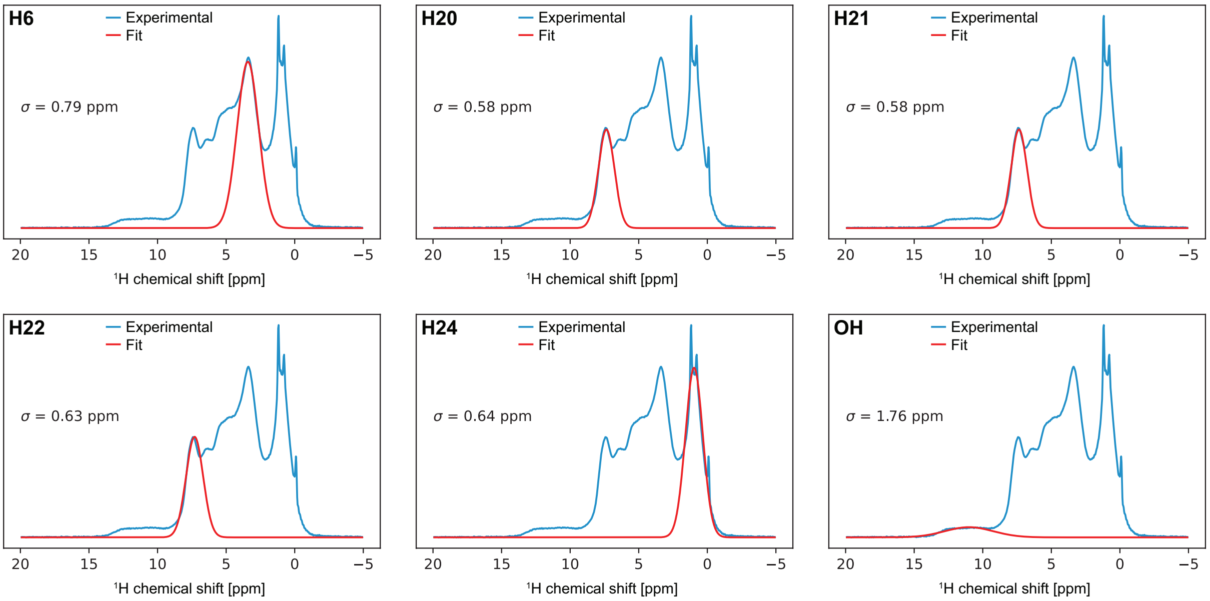

Supplementary Figure 4. Fits of individual resonances of proton sites from the 1D ^{1}H MAS NMR spectrum of amorphous AZD4625.

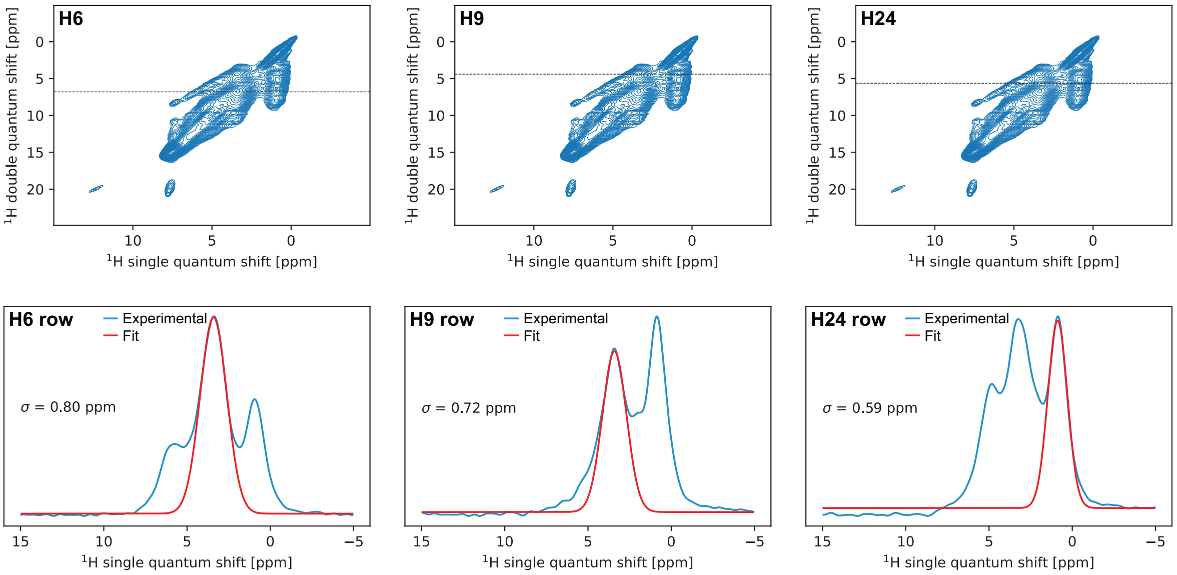

Supplementary Figure 5. Fits of individual proton resonances (bottom panels) from rows extracted from the 2D ^{1}H-^{1}H DQ/SQ MAS NMR spectrum of amorphous AZD4625 (top panels, indicated by dashed black lines).

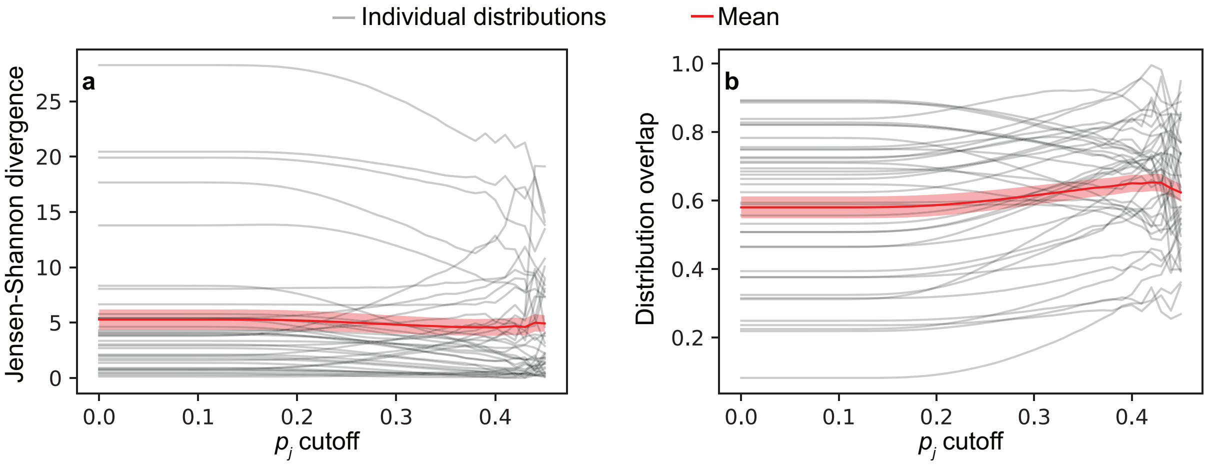

Supplementary Figure 6. (**a**) Jensen-Shannon divergence and (**b**) overlap between experimental chemical shift distributions and those obtained from the NMR ensemble. The overlap is defined as the integral under the point-wise minimum between the experimental and NMR-selected shift distribution, where each distribution is assumed to be Gaussian and with an integral of one.

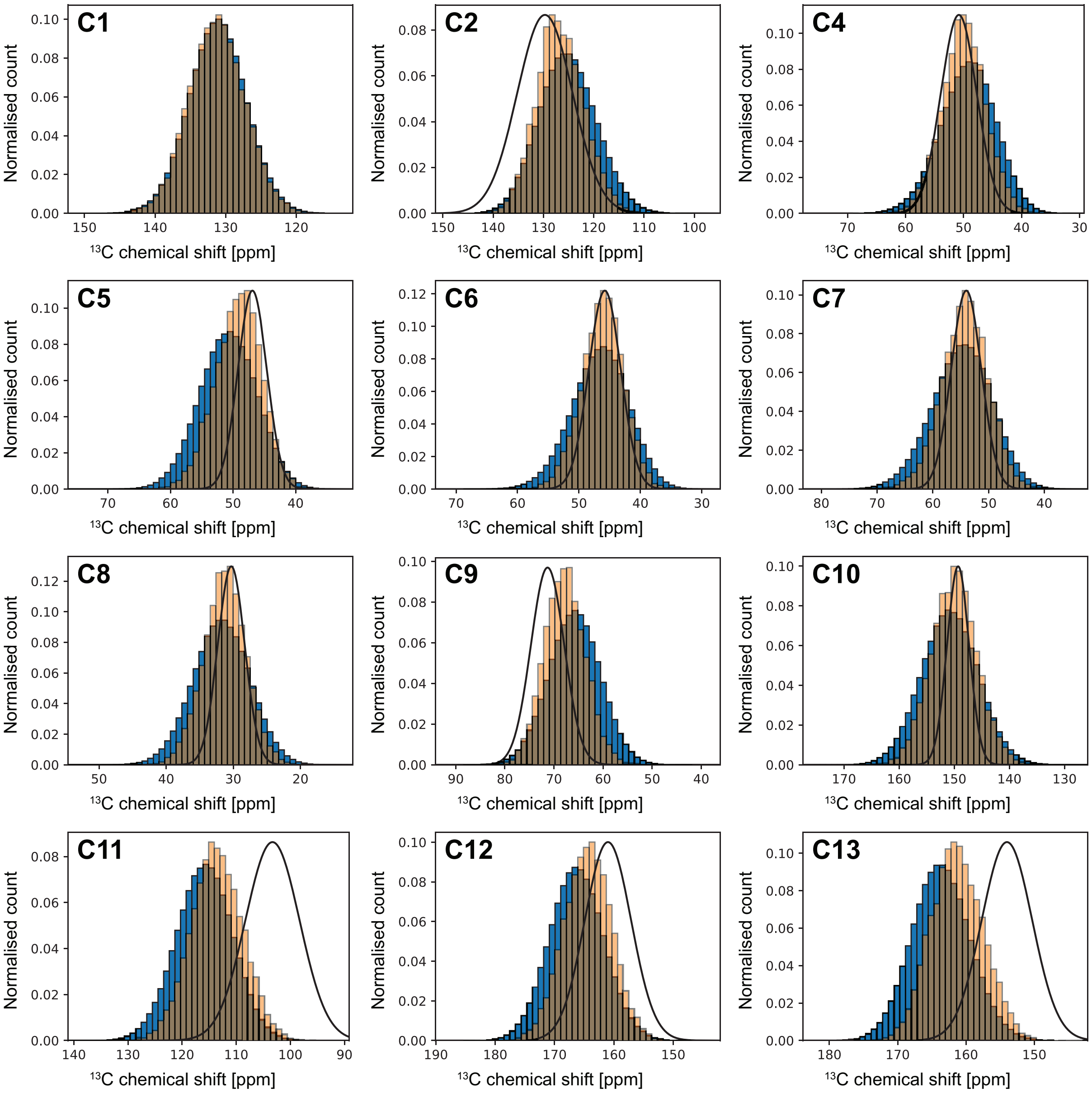

Supplementary Figure 7. Histograms of chemical shifts for individual carbons in the MD (blue) and NMR (orange) ensembles, compared to the experimental distributions when determined (black lines).

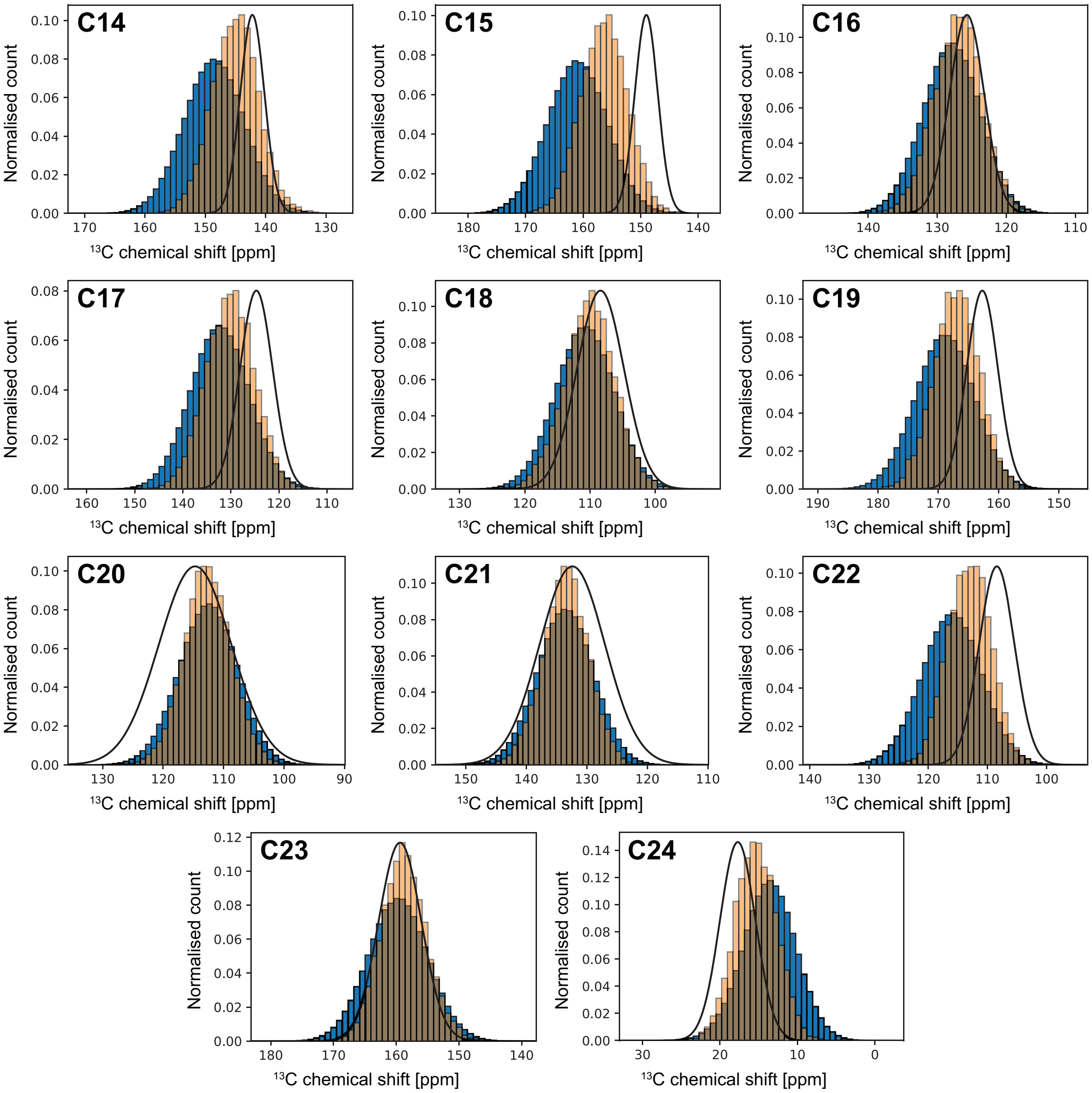

Supplementary Figure 8. Histograms of chemical shifts for individual carbons in the MD (blue) and NMR (orange) ensembles, compared to the experimental distributions when determined (black lines).

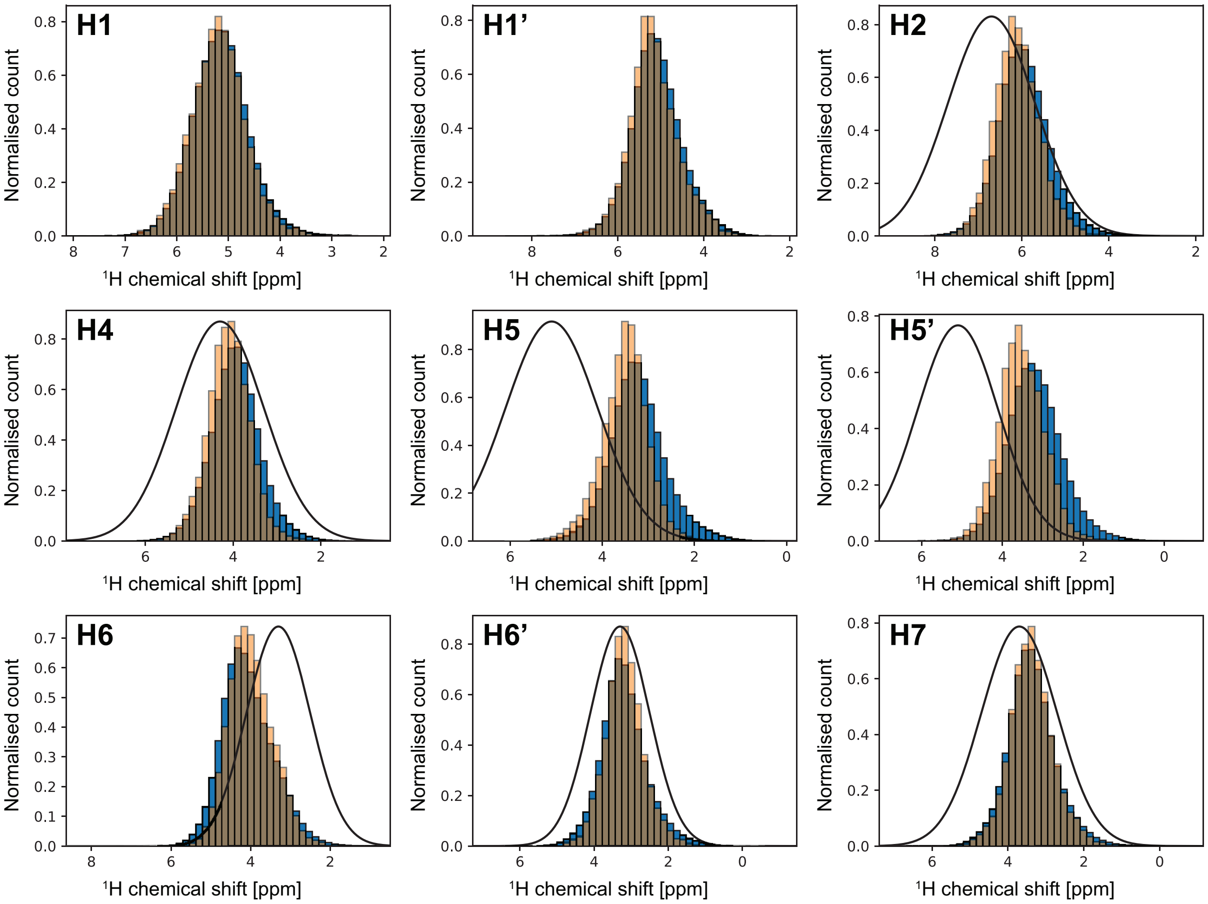

Supplementary Figure 9. Histograms of chemical shifts for individual protons in the MD (blue) and NMR (orange) ensembles, compared to the experimental distributions when determined (black lines).

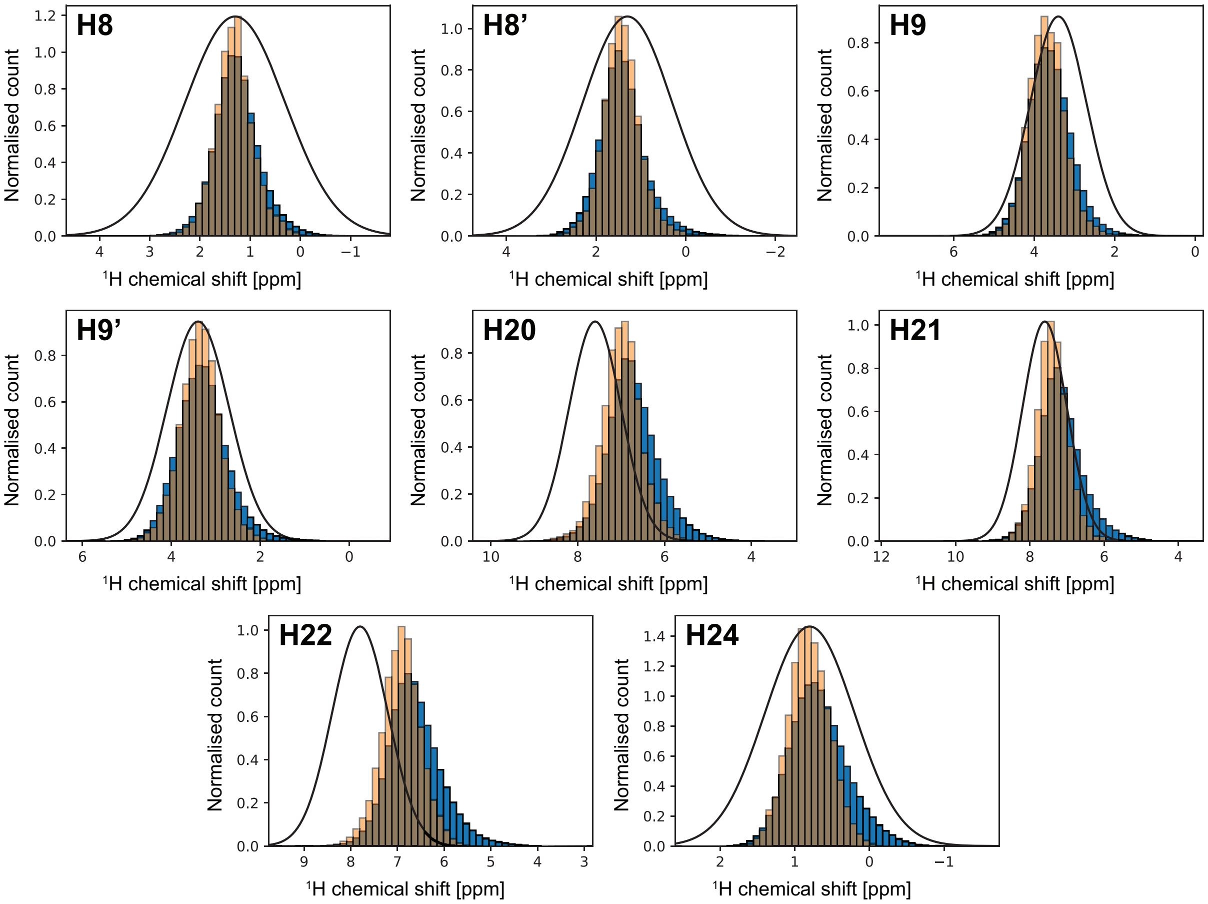

Supplementary Figure 10. Histograms of chemical shifts for individual protons in the MD (blue) and NMR (orange) ensembles, compared to the experimental distributions when determined (black lines).

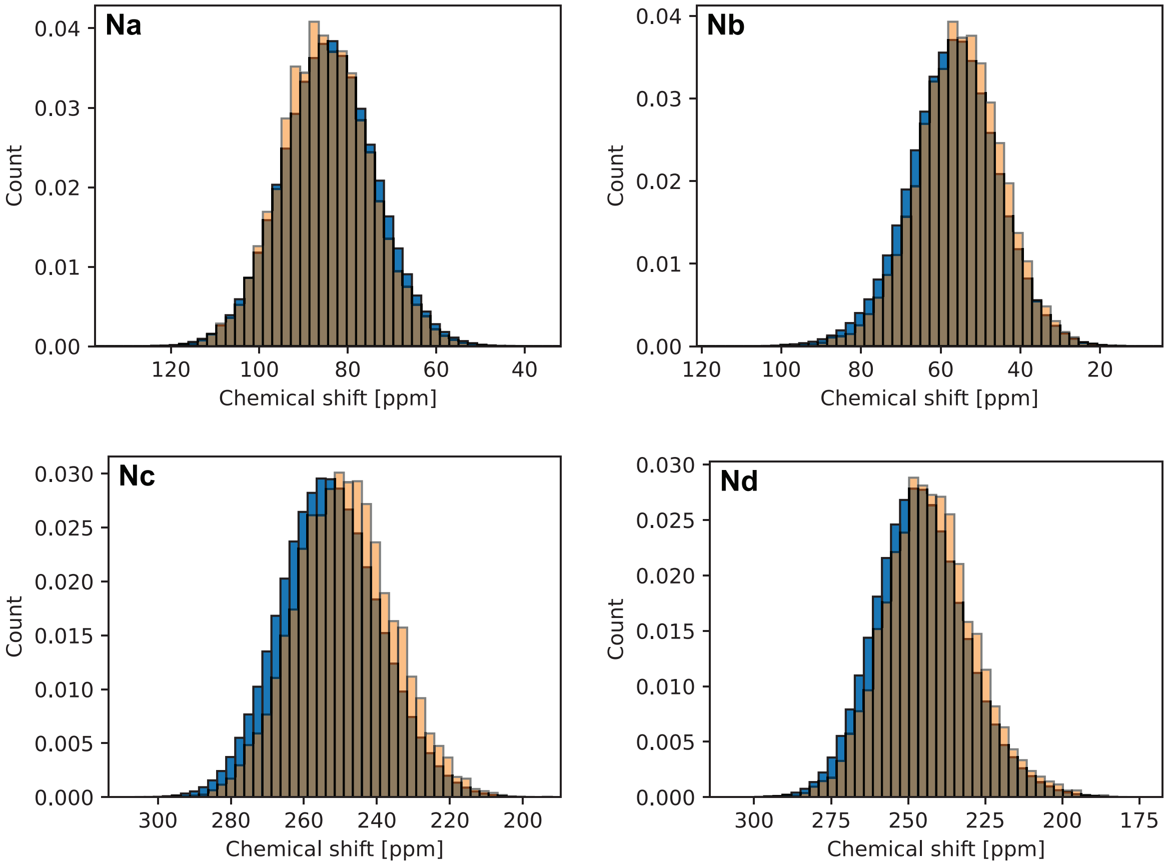

Supplementary Figure 11. Histograms of chemical shifts for individual nitrogens in the MD (blue) and NMR (orange) ensembles.

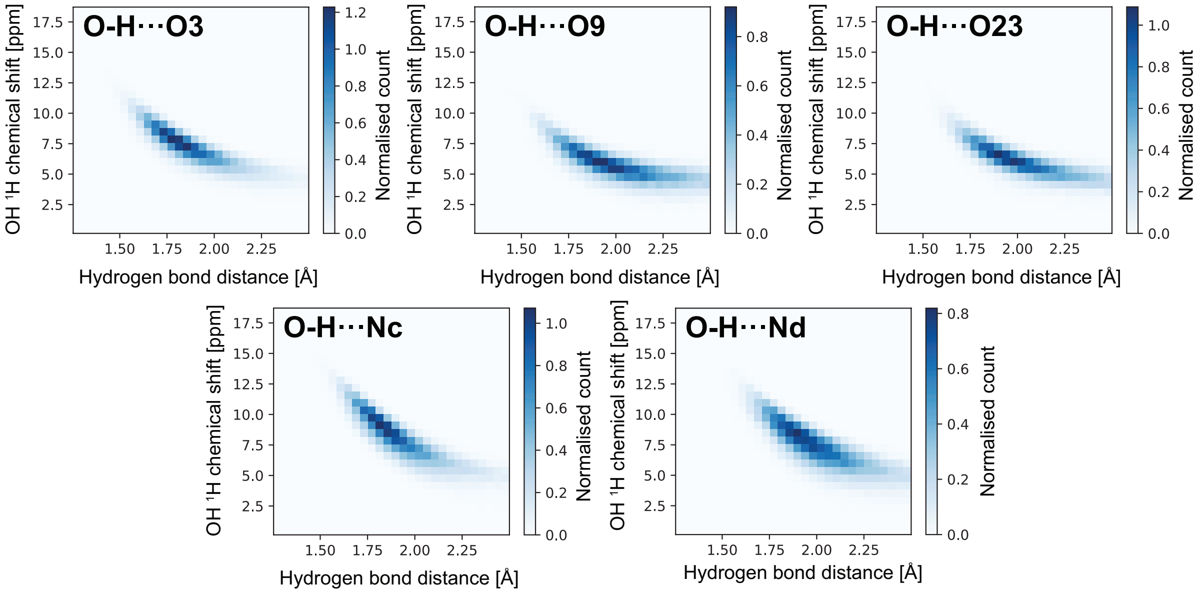

Supplementary Figure 12. Two-dimensional histograms of hydrogen bonding (H···X) distances and OH proton chemical shifts for different hydrogen bond acceptors X.

Supplementary References

1 Pines, A., Gibby, M. G. & Waugh, J. S. Proton-enhanced NMR of dilute spins in solids. *The Journal of Chemical Physics* **59**, 569-590 (1973).

2 Wu, X. L. & Zilm, K. W. Complete Spectral Editing in CPMAS NMR. *Journal of Magnetic Resonance, Series A* **102**, 205-213 (1993).

3 Fung, B. M., Khitrin, A. K. & Ermolaev, K. An Improved Broadband Decoupling Sequence for Liquid Crystals and Solids. *Journal of Magnetic Resonance* **142**, 97-101 (2000).

4 Bax, A., Freeman, R. & Frenkiel, T. A. An NMR technique for tracing out the carbon skeleton of an organic molecule. *Journal of the American Chemical Society* **103**, 2102-2104 (2002).

5 Lesage, A., Auger, C., Caldarelli, S. & Emsley, L. Determination of Through-Bond Carbon−Carbon Connectivities in Solid-State NMR Using the INADEQUATE Experiment. *Journal of the American Chemical Society* **119**, 7867-7868 (1997).

6 Marion, D., Ikura, M., Tschudin, R. & Bax, A. Rapid recording of 2D NMR spectra without phase cycling. Application to the study of hydrogen exchange in proteins. *Journal of Magnetic Resonance (1969)* **85**, 393-399 (1989).

7 Saalwächter, K., Lange, F., Matyjaszewski, K., Huang, C.-F. & Graf, R. BaBa-xy16: Robust and broadband homonuclear DQ recoupling for applications in rigid and soft solids up to the highest MAS frequencies. *Journal of Magnetic Resonance* **212**, 204-215 (2011).

8 Elena, B., de Paëpe, G. & Emsley, L. Direct spectral optimisation of proton–proton homonuclear dipolar decoupling in solid-state NMR. *Chemical Physics Letters* **398**, 532-538 (2004).

9 Becke, A. D. Density-Functional Thermochemistry .3. The Role of Exact Exchange. *J Chem Phys* **98**, 5648-5652 (1993).

10 Lee, C. T., Yang, W. T. & Parr, R. G. Development of the Colle-Salvetti Correlation-Energy Formula into a Functional of the Electron-Density. *Phys Rev B* **37**, 785-789 (1988).

11 Vosko, S. H., Wilk, L. & Nusair, M. Accurate Spin-Dependent Electron Liquid Correlation Energies for Local Spin-Density Calculations - a Critical Analysis. *Can J Phys* **58**, 1200-1211 (1980).

12 Stephens, P. J., Devlin, F. J., Chabalowski, C. F. & Frisch, M. J. Ab Initio Calculation of Vibrational Absorption and Circular Dichroism Spectra Using Density Functional Force Fields. *The Journal of Physical Chemistry* **98**, 11623-11627 (2002).

13 Grimme, S., Antony, J., Ehrlich, S. & Krieg, H. A consistent and accurate ab initio parametrization of density functional dispersion correction (DFT-D) for the 94 elements H-Pu. *The Journal of Chemical Physics* **132** (2010).

14 (Gaussian 16, Revision C.01, Gaussian, Inc., Wallingford CT, 2016).

15 Breneman, C. M. & Wiberg, K. B. Determining atom-centered monopoles from molecular electrostatic potentials. The need for high sampling density in formamide conformational analysis. *Journal of Computational Chemistry* **11**, 361-373 (1990).

16 (BIOVIA, Dassault Systèmes, San Diego, 2020).

17 Akkermans, R. L. C., Spenley, N. A. & Robertson, S. H. compass iii: automated fitting workflows and extension to ionic liquids. *Mol Simulat* **47**, 540-551 (2021).

18 (Desmond Molecular Dynamics System, D. E. Shaw Research, New York, NY, 2021).

19 Lu, C. *et al.* OPLS4: Improving Force Field Accuracy on Challenging Regimes of Chemical Space. *Journal of Chemical Theory and Computation* **17**, 4291-4300 (2021).

20 Nosé, S. A Molecular-Dynamics Method for Simulations in the Canonical Ensemble. *Mol Phys* **52**, 255-268 (1984).

21 Hoover, W. G. Canonical dynamics: Equilibrium phase-space distributions. *Phys Rev A* **31**, 1695-1697 (1985).

22 Martyna, G. J., Tobias, D. J. & Klein, M. L. Constant pressure molecular dynamics algorithms. *The Journal of Chemical Physics* **101**, 4177-4189 (1994).
